# The Chemo-Gut Pilot Study: Associations between Gut Microbiota, Gastrointestinal Symptoms, and Psychosocial Health Outcomes in a Cross-Sectional Sample of Young Adult Cancer Survivors

**DOI:** 10.3390/curroncol29050243

**Published:** 2022-04-21

**Authors:** Julie M. Deleemans, Faye Chleilat, Raylene A. Reimer, Mohamad Baydoun, Katherine-Ann Piedalue, Dana E. Lowry, Jan-Willem Henning, Linda E. Carlson

**Affiliations:** 1 Department of Oncology, Cumming School of Medicine, University of Calgary, Calgary, AB T2N 4N1, Canada; julie.deleemans@ucalgary.ca (J.M.D.); klpiedal@ucalgary.ca (K.-A.P.); janwillem.henning@albertahealthservices.ca (J.-W.H.); 2 Department of Genetics, School of Medicine, Stanford University, Stanford, CA 94305, USA; faye.chleilat@stanford.edu; 3 Department of Biochemistry & Molecular Biology, Cumming School of Medicine, Faculty of Kinesiology, University of Calgary, Calgary, AB T2N 4N1, Canada; reimer@ucalgary.ca (R.A.R.); dana.lowry@ucalgary.ca (D.E.L.); 4 Faculty of Nursing, University of Regina, Regina, SK S4S 0A2, Canada; mohamad.baydoun@uregina.ca

**Keywords:** gut microbiota, cancer survivors, psychosocial, gastrointestinal health, young adults

## Abstract

Chemotherapy adversely affects the gut microbiota, inducing dysbiosis, and negatively impacts gastrointestinal (GI) and psychosocial health during treatment, but little is known about the long-term effects or how these factors are related. Methods: This cross-sectional pilot study investigated the effects of chemotherapy on the gut microbiota, GI symptoms, and psychosocial outcomes in cancer survivors aged 18–39 years old, compared to healthy controls. Gut microbial diversity and composition were assessed from stool samples using 16S rRNA gene sequencing. Results: Survivors (*n* = 17) and healthy controls (*n* = 18) participated. Mean age at diagnosis was 31 years (±5.3). Mean time off treatment was 16.9 months (±16.4). Survivors had more severe GI symptoms, poorer psychosocial health, and increased relative abundance of *Selenomondales, Veilloneliaceae*, and *Intestinibacter*. In survivors, *Lachnospiraceae, Ruminococcaceae* and *Intestinibacter* correlated with psychosocial symptoms, while diarrhea correlated positively *with Lachnospiraceae*. Results are statistically significant. Survivors ≤6 months post-treatment had lower alpha diversity than survivors >6 months post-treatment (*p* = 0.04) and controls (*p* = 0.19). Conclusion: This small exploratory study demonstrates potential long-term gut microbial dysbiosis in cancer survivors, which may be associated with psychosocial symptoms. Larger trials concurrently and longitudinally examining gut microbiota, GI symptoms, and psychosocial outcomes are needed.

## 1. Introduction

The gastrointestinal (GI) tract harbors a complex community of bacteria, fungi, viruses, archaea, and their genomes, which affect human health and disease [1]. Dysbiosis, or a disruption of the gut microbiota, refers to adverse changes in the microbiota associated with disturbed homeostasis and disease [2]. Gut dysbiosis has been associated with behavioral changes and impaired immune function characterized by increased proinflammatory biomarkers in both humans and rodents [3,4,5]. Cancer treatments, notably chemotherapy, while lifesaving, have been associated with gut microbial dysbiosis [6,7,8]. Moreover, gut microbial associated changes in psychological and cognitive function following chemotherapy treatment have also been found [6,9,10]. Notably, while some of the studies discussed herein used techniques such as PICRUSt to infer the metabolic capacity of the microbiome, we focused our discussion of the findings from these studies on the taxonomic characterization of the microbiota using 16S rRNA sequencing, and, therefore, refer to microbiota throughout the manuscript as opposed to the microbiome.

### 1.1. Chemotherapy Impacts the Gut Microbiota

The microbiota–gut–brain axis facilitates crosstalk between the microbiota and brain via neural, endocrine, immune and metabolic pathways [1,11], which are susceptible to chemotherapy-related toxicities. In a pre-clinical study with adult female mice, 6 cycles (i.e., one treatment per every other day) of paclitaxel chemotherapy altered gut microbiota composition, which was associated with changes in taxonomic relative abundance, colonic tissue integrity, and microglial activation [12]. In clinical studies, chemotherapy treatment has been shown to alter the gut microbiota. In a sample of 36 pediatric patients with acute lymphoblastic leukemia, stool samples collected at baseline and days 3 (mid-treatment) and 7 (immediately post-chemotherapy) showed that high-dose methotrexate chemotherapy significantly reduced the overall taxonomic abundance of the gut microbiota during treatment and relative to healthy controls [7]. Another study found that chemotherapy decreased abundance of microbes belonging to the Firmicutes phylum [8]. Moreover, chemotherapy-induced mucositis, a common GI symptom in patients undergoing chemotherapy, is associated with increased symptoms of pain, anxiety, and depression [13].

### 1.2. Chemotherapy Compromises the GI Tract

The microbiota–gut–brain axis influences immune function and behavior [14]. The epithelial layer that lines the GI tract is a fine-tuned interface between the host and the external environment. The epithelia’s paracellular permeability under healthy physiological conditions is tightly regulated by tight junction proteins that prevent passage of macromolecules [15]. Chemotherapy may compromise the protective epithelial layer in the gut [12]. Compromising this layer can lead to increased intestinal permeability (i.e., “leaky gut”), which will provide easy passage of bacteria and their byproducts, such as lipopolysaccharide (LPS), a bacterial endotoxin component of the cell wall of Gram-negative bacteria, from the gut lumen into the bloodstream [9,12,16]. A compromised gut can lead to an immune response, characterized by the expression of sickness behaviors (e.g., lethargy, anxiety, cognitive dysfunction) [17]. Chemotherapy has thus been implicated in increased intestinal wall permeability, mucositis, sickness behaviors, and changes in the gut microbiota [6,7,8,9,12]. However, it is unclear whether these adverse changes persist in the longer term after treatment has ended.

### 1.3. Chemotherapy Impacts Psychosocial Health

Chemotherapy is associated with acute and chronic adverse changes in psychosocial health, but potential underlying mechanisms require elucidation. Mental health, a dimension of psychosocial health (i.e., psychological, behavioral, emotional, and social factors), in this context, refers to one’s emotional, psychological, and social well-being [18]. A growing body of research implicates the gut microbiota in symptoms of anxiety and depression [16,19,20], post-traumatic stress disorder (PTSD) [21], fatigue [22,23], cognitive and social function, pain [1,24,25], and GI disturbance [26,27,28]. Moreover, changes in the gut microbiota have been associated with anxiety and depressive behaviors in animals and humans [16,29,30,31,32].

Chemotherapy has been associated with cognitive and psychological impairments [9,33,34]. Many survivors of cancer have been treated with toxic anti-cancer therapies and face chronic physical and psychosocial challenges because of cancer and its treatments. During and after chemotherapy treatment, cognitive impairments (e.g., attention, concentration, processing speed) are reported [33]. Survivors also tend to report higher incidence of anxiety, depression, cancer-related fatigue, and other psychosocial symptoms compared to healthy peers [35,36]. However, potential underlying mechanisms that may contribute to the maintenance of psychosocial symptoms require clarification.

Although many cancer survivors face post-treatment challenges regardless of their age, young adults may face additional challenges and unmet needs [37,38]. Young adults may be particularly susceptible to psychosocial issues following a cancer experience due to the added stress of developmental challenges (e.g., finding a long-term partner, fertility concerns, career development, gaining financial stability) compared to older survivors [38,39]. Understanding potential chronic effects of cancer treatments on the gut microbiota, GI, and psychosocial outcomes, and how these factors may relate, is needed to improve cancer survivors’ wellbeing.

### 1.4. Present Study

This study investigated whether cancer treatment-related factors, specifically chemotherapy and time off treatment, were associated with gut microbiota, GI symptoms, and psychosocial outcomes in a cohort of young adult cancer survivors compared to healthy volunteers. Our study was unique from previous work as it was conducted exclusively with young adults, while most other studies investigated the gut microbiota in older adult cancer cohorts. Moreover, we specifically focused on the effects that chemotherapy had on the gut microbiota after treatment, not during treatment, and this is the only study that has comprehensively measured the gut microbiota, GI symptoms, and psychosocial outcomes using a battery of standardized questionnaires within a single study.

Previous work performed by Deleemans et al. [40] depicts the theoretical model we developed to explain chemotherapy-induced dysbiosis of the microbiota–gut–brain axis. The original study protocol was previously published [40], and the present study was modified due to the COVID-19 pandemic, which affected research operations. As such, our study was a virtual, home-based, cross-sectional pilot design, in recognition of the smaller numbers likely to be recruited and the novel data collection method. In the original study protocol, we planned to collect blood samples for cytokine and neurotransmitter analysis and to assess patients at three time points within the first 6 months post-treatment. However, this was no longer feasible due to the virtual, home-based approach we transitioned to. As such, this pilot study was not able to assess relationships between cytokines, the gut microbiota and psychosocial health. For practical reasons, this study also extended the post-treatment timeline up to 5 years after anti-cancer therapies, resulting in considerable variance in time off treatment. We also attempted to recruit another group of survivors who had received other treatments (e.g., surgery) but no chemotherapy; however, as we had to rely on convenience sampling since we could not recruit in clinic due to pandemic-related restrictions, we were not able to recruit any survivors to that group.

This study aimed to: (i) determine the feasibility of conducting this study using a virtual, home-based approach, as determined by participant accrual, consent, and retention and data completeness; (ii) investigate relationships between cancer treatment, specifically chemotherapy, time off treatment, and gut microbiota alpha diversity (primary outcome) in cancer survivors and healthy peers; (iii) compare taxonomic composition (secondary outcome) between survivors and controls; (iv) compare outcomes for GI symptoms (i.e., gas and bloating, belly pain, constipation, and diarrhea) and psychosocial health (i.e., anxiety, depressive symptoms and function, PTSD symptoms, fatigue, social isolation, pain behavior, and cognitive function) between cancer survivors and controls; (v) investigate associations between gut microbiota alpha diversity, taxonomic composition, GI symptoms, and psychosocial outcomes in survivors and healthy controls.

## 2. Materials and Methods

### 2.1. Participants

This study was approved by the Health Research Ethics Board of Alberta Cancer Committee (HREBA.CC-19-0018). Participants were recruited remotely from the Tom Baker Cancer Center in Calgary, Canada and via social media and local support and advocacy groups. Survivors were individuals between 18 and 39 years of age, diagnosed with a blood cancer or solid tumor at any stage (i.e., I–IV), had received chemotherapy, and were within 5 years from their last/final chemotherapy treatment. Survivors were ineligible if they had a diagnosis of central nervous system or colorectal cancer or had received an allogenic stem cell transplant. Healthy volunteers from the community included individuals who were currently 18 to 39 years old, had no personal previous diagnosis of cancer or irritable bowel syndrome/disease, and no current diagnosis of or treatment for anxiety, depression, or cognitive impairment (e.g., autism, attention deficit hyperactivity disorder (ADHD)). All participants had to be fluent in English and have access to a computer, smartphone or tablet with internet access.

### 2.2. Sample Size and Power Calculation

As this was a pilot study involving new recruitment and testing methods and a COVID-related adaptation of a larger protocol that had to be conducted within a proscribed timeframe, we aimed to recruit 20 survivors and 20 controls. This is consistent with previous studies investigating relationships between gut microbiota and psychosocial outcomes in people with cancer [8,23]. With this sample size (*N* = 40), we could detect medium-sized group differences (Cohen’s d = 0.5, estimated with a power of 0.80, alpha = 0.05) with an observed power of 0.46, using a one-sided Student’s t-test with the primary hypothesis that cancer survivors will have lower alpha diversity compared to controls [41].

### 2.3. Demographics and Clinical Data

Clinical and demographic variables such as current age, age at diagnosis, cancer diagnosis, stage, treatments received, history of treatment-related mucositis (for survivors), and corticosteroid medication, antibiotic, and probiotic use within the last 2 years were collected. Data on body mass index (BMI), waist-to-hip ratio (WHR), smoking, birth delivery mode (i.e., caesarian section vs. vaginal), and breastfeeding during infancy were also collected, as these variables are known to impact the gut microbiota long-term.

### 2.4. Procedure

Participants were contacted by phone to verify inclusion criteria. Written informed consent was obtained from all participants involved in the study. Following this, a time and date were scheduled for their study kits to be delivered to their homes. The kit contained all materials needed to collect stool samples at home. Participants also received a link via email to complete their questionnaires online using Remote Electronic Data Capture (REDCap). REDCap is a free, secure, browser-based application designed to support electronic data capture (EDC) for research studies provided through the Clinical Research Unit (CRU) in the University of Calgary Cumming School of Medicine. All participants received a $10 gift card in appreciation for their time and participation. Additionally, as part of our commitment to knowledge translation, each participant received a profile of their personal results (e.g., gut microbiota composition, psychosocial outcome scores, etc.), which included interpretation of their results and resources for further learning and support.

### 2.5. Psychological and Gastrointestinal Outcomes

Full details of patient-reported outcomes used were previously published [40]. Briefly, PTSD symptoms were measured using the Impact of Life Event Scale [42]. Depression, anxiety, pain behavior, fatigue, cognitive function, social isolation, and gastrointestinal (GI) outcomes of constipation, diarrhea, gas/bloating, and abdominal pain were measured via the National Institutes for Health (NIH) Patient-Reported Outcomes Measurement Information System (PROMIS) [43]. PROMIS is a set of person-centered measures that evaluates physical, mental, and social health, and is a valid measure of psychosocial and physical health outcomes among people with cancer and GI disorders [44,45,46].

### 2.6. Gut Microbiota Profiling and Analysis

Participants were instructed to use the investigator-provided stool collection kit to collect a stool sample at home. The sample was placed in a sterile conical tube, placed in a biohazard bag, and stored in the participant’s freezer until the date and time of the pre-determined pick-up from their home (not more than 3 days from time of collection). Samples were picked up by research personnel and immediately transported on ice to the University of Calgary Faculty of Kinesiology and stored at −80 °C degrees until analysis. Gut microbial profiling was conducted as previously described [47,48]. Briefly, bacterial DNA was extracted from ~60 mg of fecal matter according to the manufacturer’s instructions, using the FastDNA Spin Kits for feces (MP Biomedicals, Lachine, QC, Canada). Bacterial DNA was then diluted to 4 ng/uL using the PicoGreen DNA quantification kit (Invitrogen, Carlsbad, CA, USA) and stored at −20 °C until sequencing. Microbial composition was assessed using the MiSeq Illumina platform, which amplified the V3 and V4 regions of the 16S rRNA gene (Illumina, San Diego, CA, USA). Extracted bacterial DNA samples were sequenced at the University of Calgary’s Centre for Health Genomics and Informatics (Calgary, AB, Canada).

### 2.7. Bioinformatics and Data Analysis

All 16S rRNA gene sequence processing and analysis was conducted using R version 4.1.2. Raw bacterial DNA sequence reads were processed using the R package DADA2 (version 1.22.0) [49]. Primers were identified and removed from demultiplexed paired-end fastq files sequenced by Illumina using the primer and adaptor removal tool, cutadapt (version 2.3) [50]. Low quality sequence reads were removed and trimmed to consistent lengths using the FilterAndTrim function. Truncation criteria were as follows: forward reads truncated at position 245 bp; reverse reads at 160 bp. After truncation, error rates were estimated and later used by the dada2 algorithm. Samples were then dereplicated, which combined all identical sequencing reads and provided an abundance of said reads. Samples were then inferenced using both the error model and the dereplicated sequences. Low-quality sequences were denoised. Denoised reads were merged using the mergePairs command.

A sequence table of amplicon sequence variants was generated, and chimeras were removed (proportion of non-chimeric amplicon sequence variants (ASV) was 97.9%). ASV taxonomic classification was assigned using the assignTaxonomy and assignSpecies functions using the Silva database (version 138.1) [51]. Using the DECIPHER (version 2.22.0) [52] and phangorn (version 2.8.1) packages, a phylogenetic tree was constructed to relate the inferred sequence variants. To analyze and graphically present the denoised ASVs, the phyloseq package was used (version 1.38.0) [53]. Alpha diversity was estimated using Chao1 and Shannon indices of diversity. Significance was determined using an independent samples *t*-test. Beta diversity was estimated using a principal coordinate analysis with Bray–Curtis distances. A permutational multivariate analysis of variance (PERMANOVA) was used to determine significant differences in beta diversity between groups. Differential taxonomic abundance was analyzed using DESeq2 [54].

Descriptive statistics and frequencies were used to determine whether the virtual home-based study approach was feasible based on whether the target *N* = 40 was achieved as well as consent, retention, and completeness of data. We then investigated relationships between cancer treatment and gut microbiota alpha diversity. Categories of time off treatment were created based on the original study protocol [40], which sought to examine outcomes in only the first 6 months post-treatment. Relationships between treatment factors and alpha diversity were explored using one-way analysis of variance (ANOVA) with alpha diversity as the dependent variable and group (i.e., 3 levels: survivors ≤6 months post-treatment, >6 months post-treatment, or control) as the independent variable. Data are presented using standard error of the mean (SEM) and confidence intervals (C.I.). Gut microbiota taxonomic composition (secondary outcome) between cancer survivors and controls was determined using beta diversity and differential taxonomic abundance analyses as described above.

Outcomes for GI symptoms and psychosocial health between cancer survivors and healthy controls were explored using descriptive statistics, frequencies, independent samples, one-sided t-tests with group (2 levels: survivors or controls) as the independent variable. For PTSD symptoms and pain behavior, participants who reported no symptoms (i.e., had a score of 0) were removed from analysis for that specific variable. Anxiety, depressive symptoms and interference with function, PTSD symptoms, fatigue, social isolation, pain behavior, and cognitive function were the dependent variables for psychosocial health. Belly pain, gas and bloating, constipation, and diarrhea were the dependent variables for GI symptoms.

Lastly, we investigated associations between gut microbiota alpha diversity, taxonomic composition, GI symptoms, and psychosocial health outcomes in cancer survivors. Pearson product-moment correlations were conducted for alpha diversity analyses. Spearman’s rho was used for taxonomic composition, GI, and psychosocial outcome correlations. Only the first 60 most abundant ASVs with *n* > 10 cases were used for analysis [55]. Statistical analyses were completed using IBM SPSS version 28, with alpha set at 0.05. As this was a small exploratory study, corrections for multiple comparisons were not conducted. Hence, we recognize the elevated risk for type I errors and the need for future confirmatory studies.

## 3. Results

### 3.1. Aim 1: Study Feasibility and Participant Characteristics

A total of 54 people were screened for participation between October 2019 to January 2020, and again from January 2021 to April 2021. The pause in screening and recruitment between February 2020 to December 2020 was due to research restrictions imposed during the COVID-19 pandemic. Significant adjustments to our data collection methods were made before the study resumed. In total, 36 people were eligible for the study, and 100% consented to participate. Only one survivor dropped out due to moving away before the study began, resulting in a 97.2% retention rate. The study showed high data completion rates, with all participants (100%) providing stool samples and completing their psychosocial outcome measures. Due to protocol changes resulting from the COVID-19 pandemic, some GI (*n* = 4, 11.4%) outcome measures were not completed by people who had participated in the study prior to these changes.

A total of *N* = 17 survivors and *N* = 18 healthy controls participated in this study. Table 1 contains participants’ demographic characteristics. Percentages, means (M), and standard deviations (SD) are presented. Mean current age in years was 32.0 (SD = 5.8) for cancer survivors and 28.3 (SD = 5.3) for controls. Nearly half (45.7%) of participants were male, and about two-thirds were white (68.6%). Almost half of the survivors (47.1%) and most controls (83.3%) had undergraduate-degree level education or higher. Over half (58.9%) of survivors and controls (55.4%) earned annual income of CDN 50,000.00 or more, and just over half (58.8%) of survivors and 44.5% of controls were married or in common-law relationships and lived with their partner (58.8% of survivors, 55.6% of controls), and all except one control participant resided in a metropolitan area.

Table 2 contains the clinical and treatment-related characteristics of cancer survivors (*N* = 17) in this study. Mean age at diagnosis was 30.1 (SD = 6.0) years old. Mean time off treatment was 16.9 (SD = 16.4) months, and nearly half (41.2%) of survivors had completed treatment within the last 6 months, while 58.8% completed treatment between 11 months to 5 years prior to study participation. About two-thirds (64.7%) of survivors had a hematological cancer diagnosis. Survivors were diagnosed at stages I through IV, although nearly half (47.0%) were diagnosed with more advanced (stage III or IV) cancers. All survivors had previously received chemotherapy. About one-third (35.3%) had also received surgery, 17.6% had received radiation therapy, but few had received hormone (11.8%) or immunotherapy (5.9%). Almost one-quarter (23.5%) reported experiencing mucositis either during or after cancer treatments.

In the 2 years prior to the study, compared to controls (38.9%), fewer survivors (23.5%) reported using probiotics, about half of survivors (52.9%) and controls (50.0%) reported using antibiotics, and nearly one-quarter (23.5%) of survivors used corticosteroid medications. Mean body mass index (BMI) was 25.6 (SD = 6.0) for survivors and 24.4 (SD = 2.8) for controls. Mean waist to hip ratio (WHR), a measure of fat distribution and a potential marker of metabolic health [56], was 0.85 (SD = 0.1) for survivors and 0.82 (SD = 0.1) for controls, with less than half (41.2%) of survivors, but most (88.9%) of controls, in the WHR low health risk category. Most survivors (94.1%) and controls (72.2%) reported no tobacco smoking. Just over half (58.8%) of survivors, but all (100%) controls, reported being breastfed as infants, and about two-thirds (64.7%) of survivors and the majority (88.9%) of controls were born vaginally.

### 3.2. Aim 2: Relationships between Cancer Treatment-Related Factors and Gut Microbiota Alpha Diversity

**Figure 1:**
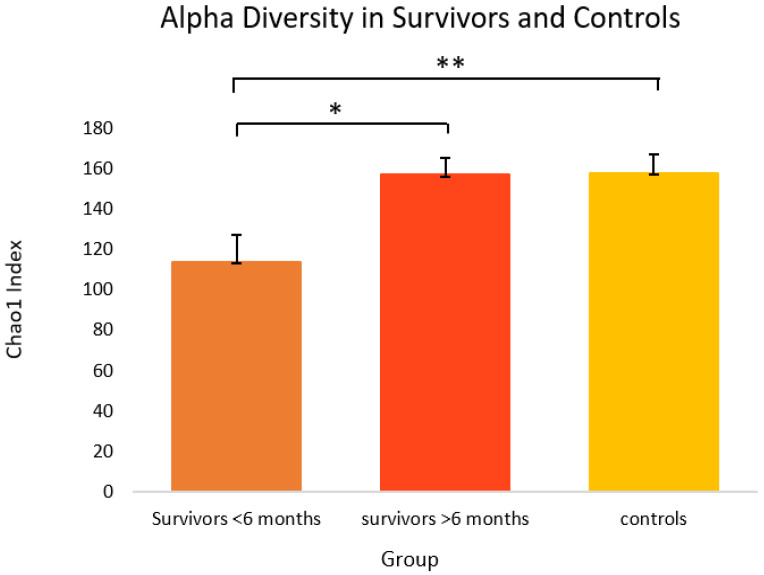
Alpha diversity on the Chao1 index in cancer survivors and healthy controls. Group means are presented. Significance is denoted by * at the *p* < 0.05 level, and ** at the *p* < 0.01 level. Error bars represent standard error of the mean (SEM). Group *n* = 7, 10, and 18, respectively.

As shown in Figure 1, when the survivor group was stratified by time off treatment (i.e., ≤6 months post-treatment versus >6 months post-treatment), one-way ANOVA revealed survivors ≤6 months post-treatment (M = 114.0, SEM = 13.4) had significantly lower alpha diversity than survivors at >6 months post-treatment (M = 157.4, SEM = 8.2) and controls (M = 158.2, SEM = 8.9) using the Chao1 index [F(2,34) = 4.56, *p* = 0.018]. Tukey’s HSD test for multiple comparisons found that the mean value of alpha diversity (Chao1) was significantly different between survivors ≤6 months post-treatment and >6 months post-treatment (*p* = 0.040, 95% C.I. = −85.03, −1.74), and survivors ≤6 months post-treatment and controls (*p* = 0.019, 95% C.I. = −81.81, −6.52). Alpha diversity on the Shannon index approached significance (*p* = 0.066) (Supplementary Figure S1).

### 3.3. Aim 3: Gut Microbiota Taxonomic Composition in Cancer Survivors and Healthy Controls

**Figure 2:**
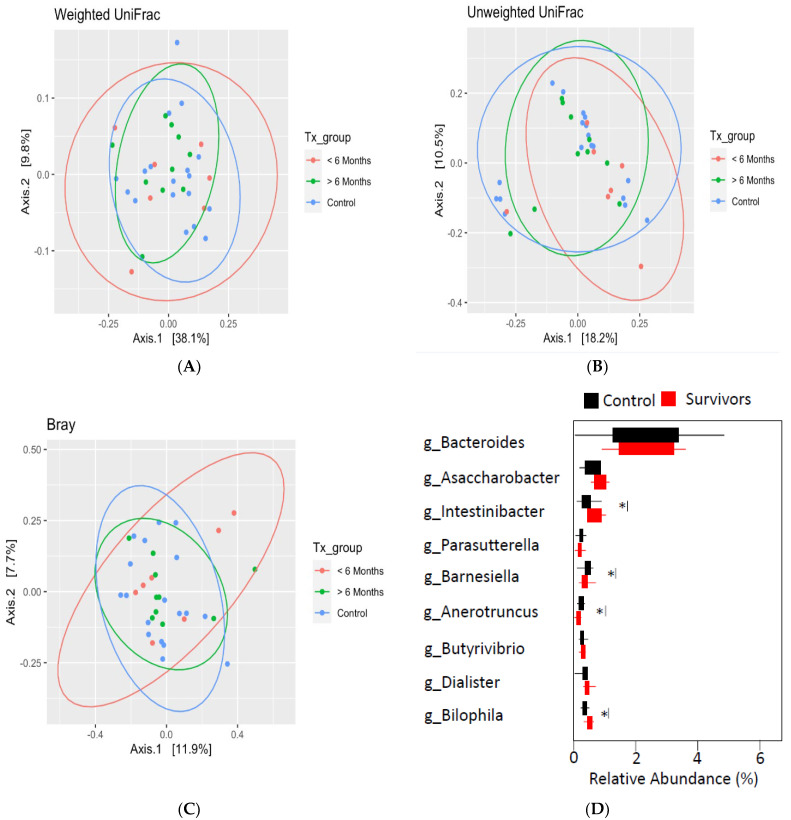
Beta diversity as determined by principal coordinate analysis with weighted (**A**), unweighted (**B**) UniFrac distances, and Bray–Curtis distance (**C**) revealed no significant differences between groups. (**D**) Differential taxonomic abundance analysis at the genus level. Significance is denoted by * at the *p* < 0.05 level.

Analysis of beta diversity using principal coordinate analysis (Figure 2A–C) with Bray–Curtis distances found no significant differences between survivors ≤6 months post-treatment, survivors >6 months post-treatment, and healthy controls. Despite the lack of differences in overall community structure, differential taxonomic abundance analysis showed significantly increased relative abundance of bacteria from *Selenomondales* (*p* < 0.05), *Veilloneliaceae* (*p* < 0.05) (data not shown), and *Intestinibacter* (*p* = 0.04) in survivors compared to controls. Moreover, decreased relative abundance of *Barnesiella* (*p* = 0.03), *Bilophila* (*p* = 0.01), and *Anaerotruncus* (*p* = 0.04) was also observed in survivors (see Figure 2D).

Since beta diversity analysis revealed no significant differences between groups, we decided that merging the survivor group for subsequent analyses was warranted, which also served to increase the power for these analyses. For the analyses of GI symptoms and psychosocial outcomes, there was insufficient evidence in the literature suggesting that time off treatment warranted stratification of the survivor group. Moreover, another study we recently completed with *N* = 317 cancer survivors showed that time off treatment did not significantly impact GI symptoms or mental health [57]. As such, we decided not to stratify the survivor group for the remainder of the analyses.

### 3.4. Aim 4: Outcomes for GI Symptoms and Psychosocial Health in Cancer Survivors and Healthy Controls

**Figure 3:**
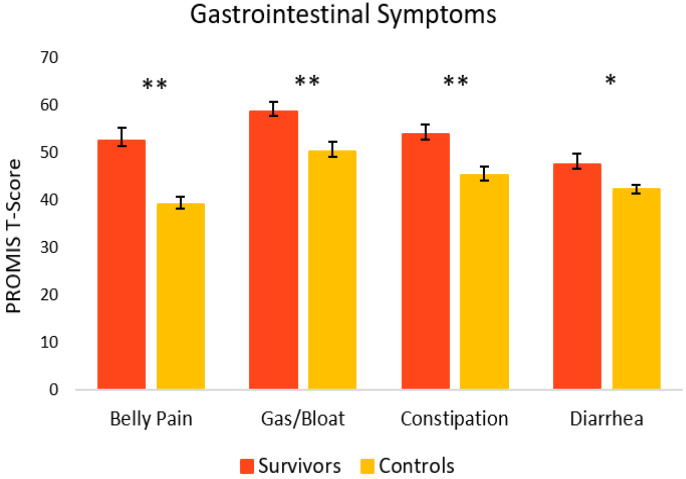
Gastrointestinal symptoms in cancer survivors and healthy controls. Group means are presented. Error bars represent standard error of the mean (SEM). Significance is denoted by ** at the *p* < 0.01 level, and * at the *p* < 0.05 level. Group *n* = 13–18.

Figure 3 depicts GI symptoms in cancer survivors and healthy controls. Overall, survivors reported significantly more GI symptoms relative to controls. Survivors reported significantly more belly pain (M = 52.5, SEM = 2.8) [t(27) = 4.46, *p* = < 0.001, 95% C.I. = 7.22, 19.50], as well as gas and bloating (M = 58.7, SEM = 1.9), [t(29) = 2.99, *p* = 0.003, 95% C.I. = 2.71, 14.40] relative to controls (M = 39.1, SEM = 1.5 and M = 50.2, SEM = 2.0, respectively). Survivors also experienced significantly more constipation (M = 53.8, SEM = 2.1) [t(29) = 3.01, *p* = 0.003, 95% C.I. = 2.77, 14.50] and diarrhea (M = 47.6, SEM = 2.2), [t(16) = 2.22, *p* = 0.042, 95% C.I. = 0.23, 10.41] compared to controls (M = 45.2, SEM = 1.9 and M = 42.3, SEM = 0.9, respectively).

**Figure 4:**
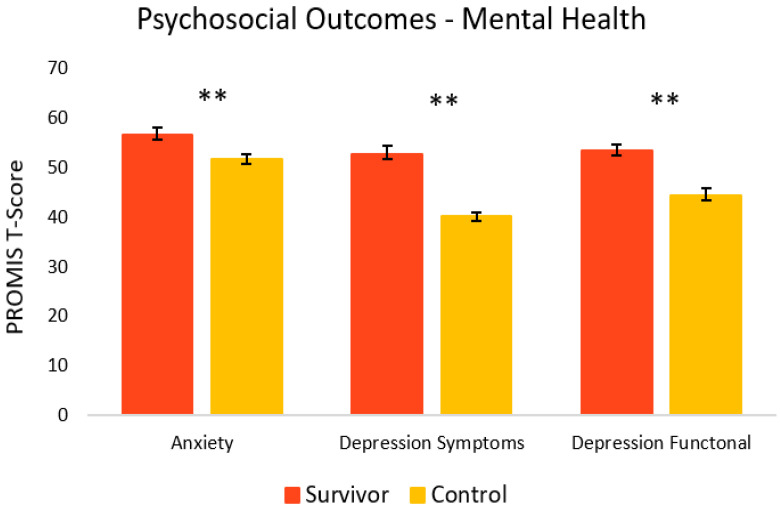
Psychosocial mental health-related outcomes in cancer survivors and healthy controls. Group means are presented. Error bars represent standard error of the mean (SEM). Significance is denoted by ** at the *p* < 0.01 level. Group *n* = 17–18.

**Figure 5:**
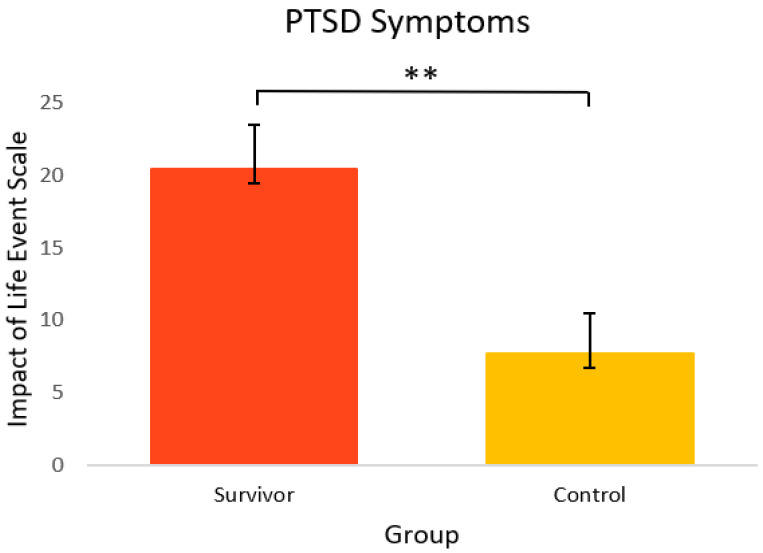
PTSD symptoms in cancer survivors and healthy controls. Group means are presented. Error bars represent standard error of the mean (SEM). Significance is denoted by ** at the *p* < 0.01 level. Group *n* = 14–10.

Figure 4 shows the mental health-related psychosocial outcomes for cancer survivors versus healthy controls. Relative to controls, survivors had significantly poorer outcomes. On average, survivors (M = 56.7, SEM = 1.4) had significantly more anxiety symptoms [t(33) = 2.86, *p* = 0.004, 95% C.I.= 1.46, 8.65] compared to controls (M = 51.6, SEM = 1.1). Survivors also had significantly more depressive symptoms (M = 52.6, SEM = 1.6) [t(23) = 6.89, *p* = <0.001, 95% C.I. = 8.69, 16.17] and depressive functional interference (M = 53.4, SEM = 1.1) [t(33) = 5.09, *p* = <0.001, 95% C.I. = 5.44, 12.68] relative to controls (M = 40.2, SEM = 0.8 and M = 44.4, SEM = 1.4, respectively). As seen in Figure 5, PTSD symptoms were also significantly higher in survivors (M = 20.4, SEM = 3.0) [t(22) = 2.98, *p* = 0.003, 95% C.I. = 3.86, 21.60] compared to controls (M = 7.7, SEM = 2.7).

**Figure 6:**
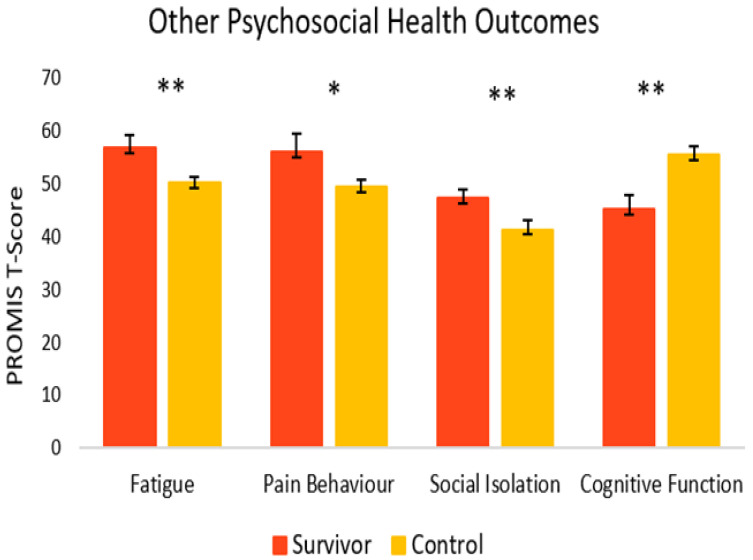
Psychosocial outcomes in cancer survivors and healthy controls. Group means are presented. Error bars represent standard error of the mean (SEM). Significance is denoted by ** at the *p* < 0.01 level, and * at the *p* < 0.05 level Group *n* = 8–18.

As seen in Figure 6, for other psychosocial health outcomes, survivors also fared worse. Compared to controls (M = 41.4, SEM = 1.7), survivors had significantly greater symptoms of social isolation (M = 47.4, SEM = 1.6) [t(33) = 2.56, *p* = 0.008, 95% C.I. = 1.22, 10.74]. Survivors also reported significantly more fatigue (M = 56.9, SEM = 2.3) [t(22) = 2.64, *p* = 0.008, 95% C.I. = 1.41, 11.81] and more pain behavior (M = 56.1, SEM = 3.4) [t(9) = 1.83, *p* = 0.05, 95% C.I. = −1.56, 14.65] compared to controls (M = 50.3, SEM = 1.0 and M = 49.5, SEM = 1.2, respectively). Survivors reported significantly lower cognitive function (M = 45.3, SEM = 2.5), [t(28) = −3.43, *p* = <0.001, 95% C.I. = −16.24, −4.09] compared to healthy controls (M = 55.4, SEM = 1.6).

### 3.5. Aim 5: Associations between Gut Microbiota Alpha Diversity, Taxonomic Composition, GI Symptoms and Psychosocial Health Outcomes in Cancer Survivors

Pearson correlation analysis revealed no significant associations between alpha diversity and GI symptoms or psychosocial outcomes in cancer survivors. However, as seen in Table 3, when groups were combined, higher alpha diversity was associated with fewer depressive functional interference symptoms (rs = −0.47 to −0.39, ps < 0.05) and higher cognitive function (rs = 0.33 to 0.34, ps < 0.05) on both the Chao1 and Shannon indexes.

**Figure 7:**
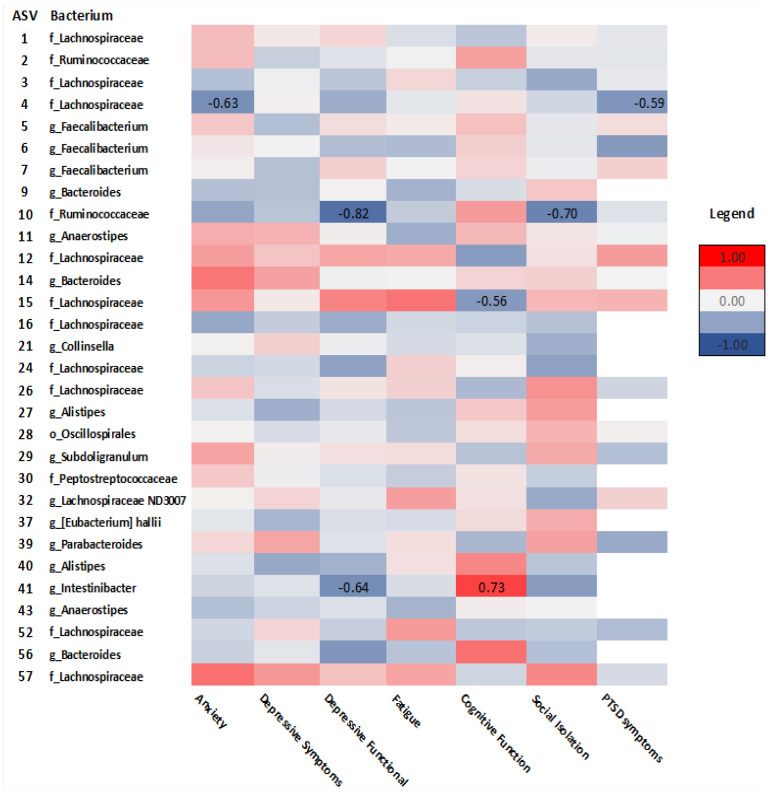
Heatmap of correlations between psychosocial outcomes and ASVs at the order (o), family (f), and genus (g) levels in cancer survivors. Spearman’s rho is presented. (Values for n range from 10–17).

**Figure 8:**
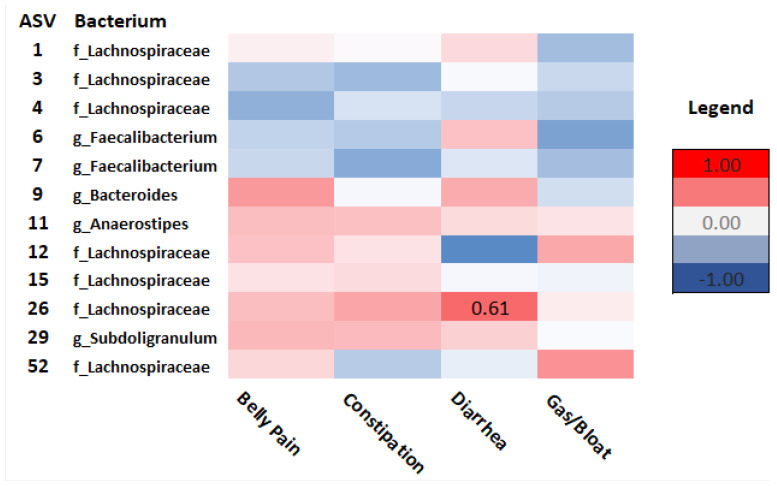
Heatmap of correlations between GI outcomes and ASVs at the order (o), family (f), and genus (g) levels in cancer survivors. Spearman’s rho is presented. (Values for n range from 10–17).

Spearman’s rho correlation analysis examined relationships between taxonomic composition and GI and psychosocial symptoms in survivors and controls. As seen in Figure 7, in survivors, *Lachnospiraceae* (ASV_4) correlated with anxiety (rho = −0.63, *p* = 0.02), PTSD symptoms (rho = −0.59, *p* = 0.05), and cognitive function (ASV_15) (rho = −0.56, *p* = 0.04). *Ruminococcaceae* (ASV_10) correlated with depressive functional interference (rho = −0.82, *p* = <.001) and social isolation (rho = −0.70, *p* = 0.01). *Intestinibacter* (ASV_41) correlated with cognitive function (rho = 0.73, *p* = 0.02) and depressive functional interference (rho = −0.64, *p*= 0.05). Diarrhea was positively correlated with *Lachnospiraceae* (ASV_26) (rho = 0.61, *p* = 0.03) (Figure 8).

In healthy controls, *Ruminococcaceae* (ASV_2) (rho = −0.50, *p* = 0.04), *Faecalibacterium* (ASV_6) (rho = −0.66, *p* =0.01), *Lachnospiraceae ND3007* (ASV_32) (rho = −0.64, *p* = 0.011), *Intestinibacter* (ASV_41) (rho = −0.56, *p* = 0.05), and *Lachnospiraceae* (ASV_54) (rho = 0.61, *p* = 0.03) all correlated with anxiety. *Ruminococcaceae* (ASV_2) (rho = −.60, *p* = 0.01) and *Lachnospiraceae* (ASV_54) (rho = 0.73, *p* = 0.01) both correlated with fatigue. *Ruminococcaceae* (ASV_10) (rho = 0.50, *p* = 0.05) and *Lachnospiraceae* (ASV_54) (rho = 0.59, *p* = 0.04) both correlated positively with depressive functional interference. *Lachnospiraceae* (ASVs 26, 54, and 57) all correlated negatively with cognitive function (rho = −0.66–−0.70, *p* < 0.05). *Bacteroides* (ASVs 8 and 56) both correlated negatively with social isolation (rho = −0.63–−0.74, *p* < 0.05). *Lachnospiraceae* (ASVs 4 and 16) (rho = 0.61–0.92, *p* < 0.05) and *Ruminococcaceae* (ASV_10) (rho = 0.82, *p* = 0.004) all correlated positively with pain behavior. *Peptostreptococcaceae* (ASV_30) correlated negatively with PTSD symptoms (rho = −0.70, *p* = 0.03). *Bacteroides* (ASV_14) was positively associated with gas and bloating (rho = 0.50, *p* = 0.05), while *Lachnospiraceae* (ASV_52) was negatively correlated with diarrhea (rho= −0.62, *p* = 0.01) (see Supplementary Figures S2 and S3).

## 4. Discussion

### 4.1. Study Feasibility

This study determined the feasibility of a virtual, home-based study design for investigating the effects of cancer treatments on gut microbiota, GI, and psychosocial outcomes in young adult cancer survivors compared to healthy controls. We found that the virtual, home-based protocol was both feasible and pragmatic. With the ongoing COVID-19 pandemic, research designs have had to explore approaches for data collection methods that differ from traditional strategies. Participants in our study seemed satisfied with the virtual, home-based approach. We expect this was likely because it allowed individuals to participate entirely from the comfort and safety of their homes, rather than having to travel to a research institution or hospital, which could cause unnecessary inconvenience and stress. Employing virtual, home-based strategies for study designs may also help to reduce barriers to study participation and overcome accessibility issues.

### 4.2. Gut Microbiota in Cancer Survivors and Healthy Peers

Relationships between cancer treatment-related factors and gut microbiota alpha diversity were observed. For cancer survivors, the mean age at diagnosis was 30 years old, and the mean time off treatment was about 1.5 years. Nearly half (41.2%) of survivors had completed their cancer treatments within the last 6 months, while 58.8% completed treatment from 11 months to a maximum of 5 years prior to participation in the study, and all survivors had received chemotherapy. Chemotherapy did not appear to impact alpha diversity within the survivor group. However, we found differences in gut microbiota alpha diversity and taxonomic composition in cancer survivors compared to healthy controls. Additionally, when the survivor group was stratified by time off treatment, gut microbiota alpha diversity in survivors ≤6 months post-treatment, but not >6 months post-treatment, was significantly lower on the Chao1 index, a measure of species richness, compared to controls.

These findings are consistent with previous research in both preclinical and clinical models showing that the relative abundance of gut microbiota tends to be lower immediately after pelvic radiotherapy and chemotherapy, compared to levels observed before anti-cancer therapies begin [6,8,58]. In a sample of 28 patients with non-Hodgkin’s lymphoma who received the same myeloablative chemotherapy conditioning regimen, compared to baseline, gut microbiota alpha diversity was significantly lower immediately after chemotherapy [8]. Furthermore, in patients who received pelvic radiotherapy, significant reductions in alpha diversity immediately after the course of treatment were found relative to controls and in patients who developed treatment-associated diarrhea, compared to patients who did not have diarrhea [58].

Our study found no significant differences in beta diversity in the ≤6 months post-treatment group compared to survivors >6 months post-treatment and healthy controls. However, differential taxonomic abundance analysis revealed that compared to controls, survivors showed increased relative abundance of bacteria from *Selenomondales, Veilloneliaceae*, and *Intestinibacter*. Decreased relative abundance of bacteria from *Barnesiella, Bilophila*, and *Anaerotruncus* was also observed in survivors. Consistent with our findings, previous studies also found decreased relative abundance of *Bifidobacterium* [7], but reduced abundance of bacteria from Firmicutes immediately following chemotherapy [8].

When examining the effects of chemotherapy on the gut microbiota and incidence of mucositis in 28 adult non-Hodgkin’s lymphoma patients, decreased abundance of Firmicutes and Actinobacteria, increased Proteobacteria, and reduced energy and vitamin metabolism were found [8]. While these studies show evidence of changes in gut microbiota abundance following cancer treatments, our study is one of the first to show that changes in gut microbiota persist beyond treatment, with lower alpha diversity and differential taxonomic abundance persisting for up to at least 6 months post-chemotherapy, but possibly longer for some survivors. Moreover, although based on a small sample size, these results may suggest that the first 6 months post-treatment may be a particularly opportune time to implement microbiota-based interventions.

### 4.3. GI and Psychosocial Symptoms

Cancer survivors’ GI symptoms and psychosocial health were also examined in comparison to healthy controls. Consistent with our hypothesis, survivors in this study experienced significantly more GI symptoms, specifically belly pain, constipation, diarrhea, and gas and bloating compared to controls. This is consistent with previous research suggesting that survivors of cancer experience GI symptoms both during and after the end of treatment [59,60]. Moreover, evidence from another recent study completed by our group found that in a sample of 317 cancer survivors, 52% reported persistent, moderate to severe GI symptoms lasting for an average of 2.5 years post-treatment. Additionally, more symptoms of constipation, diarrhea, belly pain, gas and bloating, and GI symptom severity were all significantly associated with poorer mental and physical health [57].

Evidence also suggests that the gut microbiota play a crucial role in chemotherapy and radiation therapy-induced GI symptoms by modulating intestinal epithelial integrity, repair, and immune function. A systematic review by Touchefeu et al. [28] found that in patients receiving chemotherapy and radiation therapy, significant changes in the gut microbiota occurred, which were characterized by decreases in bacteria such as *Bifidobacterium* and increased *Enterobacteriaceae* and *Bacteroides*, as well as symptoms of diarrhea and bacteremia. Interestingly, our study showed that increased *Lachnospiraceae* correlated with more symptoms of diarrhea in survivors. This finding was unexpected, since other studies have shown that *Lachnospiraceae* and *Ruminococcaceae* provide protective benefits against *Clostridium difficile* infection, characterized by severe diarrhea symptoms, in patients receiving allogenic hemopoietic stem cell transplants [61]. In our healthy controls, there was a negative correlation between *Lachnospiraceae* and diarrhea, which may suggest that GI symptoms could be influenced by unique host–microbe relationships that occur in healthy versus disease states. Nevertheless, findings from our study provide further evidence of increased, and potentially prolonged, GI symptoms in cancer survivors that may be related to dysbiosis of the gut microbiota.

Consistent with previous research, young adult cancer survivors in this study reported significantly poorer psychosocial health relative to controls. Specifically, survivors experienced more anxiety, depressive symptoms and functional interference, PTSD symptoms, fatigue, pain behavior, social isolation, and lower cognitive function. These findings align with previous work showing that cancer survivors, and young adults in particular, are at risk of experiencing psychosocial challenges following a cancer diagnosis [34,37].

However, previous studies in cancer cohorts typically did not measure both GI and psychosocial symptoms within the same study. Thus, we provide evidence that cancer survivors experience more GI symptoms and poorer psychosocial outcomes compared to healthy peers, the prevalence and intensity of which may exert bi-directional influence on each other. However, potential underlying mechanisms driving these sustained GI and psychosocial symptoms require clarification.

### 4.4. Relationships between Gut Microbiota, GI, and Psychosocial Symptoms

Our study provides compelling evidence that survivors of cancer experience more GI symptoms and poorer psychosocial health relative to healthy peers, which may be influenced, at least in part, by changes in the gut microbiota. Given this, we explored associations between alpha diversity, taxonomic composition, GI symptoms, and psychosocial outcomes in cancer survivors. In survivors, alpha diversity was not associated with GI symptoms or psychosocial outcomes. However, when groups were combined, higher alpha diversity, an indicator of gut health, was associated with fewer depressive functional interference symptoms and higher cognitive function. These findings are consistent with other studies showing that gut dysbiosis may play a role in depressive disorders and cognitive function [25,62,63].

When examining associations between GI symptoms and psychosocial outcomes and specific bacteria, we found that in survivors, lower abundance of *Lachnospiraceae* was associated with more anxiety, PTSD symptoms, and higher cognitive function. Moreover, lower *Ruminococcaceae* abundance correlated with more depressive functional interference and social isolation, while lower *Intestinibacter* abundance was associated with poorer cognitive function, but more depressive functional interference. More diarrhea symptoms were associated with higher abundance of *Lachnospiraceae*.

Interestingly, these patterns of associations differed between survivors and controls. For instance, in controls, *Ruminococcaceae* correlated positively with depressive functional interference, which is the opposite of what was seen in survivors. Further, *Lachnospiraceae* was positively correlated with anxiety, depressive functional interference, and fatigue, and negatively associated with cognitive function in controls, but under-represented in survivors, such that only 35% of survivors versus 67% of healthy peers were colonized with *Lachnospiraceae* (ASV_54). In controls, *Bacteroides* correlated negatively with social isolation, such that lower abundance of *Bacteroides* (ASV_8) was associated with more social isolation symptoms, and this bacterium was underexpressed in survivors (41%) compared to 78% in healthy controls. These associations suggest differential patterns in relationships between the gut microbiota and psychosocial health in cancer survivors and healthy peers.

Previous studies in both animal models and people with cancer have shown differences in taxonomic abundance and composition following chemotherapy treatment. However, ours is the first study to look comprehensively at several psychosocial and GI outcomes previously shown in other patient cohorts, such as irritable bowel syndrome (IBS) [27], to be associated with gut microbiota. Consistent with our findings, associations between gut microbiota and psychosocial symptoms have also been found in cancer cohorts. In a sample of 126 breast cancer survivors, Okubo et al. [10] found that relative abundance of *Bacteroides* was significantly associated with fear of cancer recurrence (FCR), a cancer-specific dimension of anxiety, and higher alpha diversity was associated with less FCR. Additionally, in survivors who had received chemotherapy, higher relative abundance of Firmicutes was associated with lower FCR scores, while higher relative abundance of *Bacteroides* was associated with greater FCR [10].

Another study with 12 breast cancer survivors found that increased fatigue interference was associated with higher Shannon diversity and frequencies of *Faecalibacterium* and *Prevotella*, while anxiety was associated with frequencies of *Coprococcus* and *Bacteroides* [20]. These findings differ from our study, which found only *Lachnospiraceae* to be negatively associated with anxiety. This may be due to different treatment protocols, as in the Paulsen et al. [20] study, in which participants were all survivors of breast cancer for which hormone therapies are a more common part of care, whereas in our study, only 2 survivors had received hormone therapy. Previous research suggests that hormones such as estrogen may also impact, or be affected by, the gut microbiota [64]. Another potential reason for this difference may be that women in the Paulsen et al. [20] study were part of a larger cohort participating in a physical activity intervention for cancer survivors. Previous research has shown that physical activity can impact the gut microbiota [5,65]; thus, it is possible that the difference in findings could also be, at least in part, due to the physical activity intervention.

Among 50 patients with rectal cancer completing both chemotherapy and radiation treatment, fatigue was associated with increased abundance of *Eubacterium, Streptococcus, Adlercreutzia*, and *Actinomyces* at the end of treatment, compared to non-fatigued patients [66]. Interestingly, in our study, no significant correlations were found between any of the bacteria and fatigue in survivors, but in healthy controls, *Ruminococcaceae* was negatively associated with fatigue, while *Lachnospiraceae* correlated positively with fatigue. Our lack of findings with respect to fatigue in survivors could be due to the fact that other studies with cancer patients used clinical cutoffs to differentiate severely fatigued patients who participated in research while on active cancer treatments [23,66]. In our study, although survivors were significantly more fatigued compared to controls, the mean fatigue score for survivors was 56.9 (SEM = 2.3), which corresponds to mild fatigue [43]. Thus, the combination of overall mild fatigue in survivors and being off treatment may have contributed to the lack of associations between fatigue and the gut microbiota in our study.

In the same cross-sectional sample of rectal cancer patients, Gonzalez-Mercado et al. [67] found that depressive symptoms correlated negatively with several types of bacteria, including *Intestinibacter*, *Lachnospiraceae*, and *Ruminococcaceae*. Similar associations were found in our study, such that *Intestinibacter* and *Ruminococcaceae* were both negatively correlated with depressive functional interference in survivors. Evidence from patients with Crohn’s disease suggest that elevated levels of *Intestinibacter* may impact inflammatory processes and GI symptomology [68]. However, potential mechanisms mediating associations between behavioral outcomes and the gut microbiota require further exploration.

Previous research and results from the present study thus highlight the psychosocial challenges that some cancer survivors face and provide compelling evidence for a potential link between psychosocial symptoms and gut microbiota in people with cancer. However, further research is needed to clarify the functional role of these microbiota, in addition to other bioinformatic strategies such as quantification of microbial metabolites, inflammatory, and hypothalamic-pituitary-adrenal (HPA)-axis-related biomarkers.

### 4.5. Limitations

Key limitations of the present study include a small sample size, diverse cancer types and treatment regimens for cancer survivors, inability to assess other biomarkers such as cytokines, no controlling for multiple comparisons that may increase type I error, and a potential lack of generalizability to other patient cohorts (e.g., colorectal cancer, older adults). While our sample size was relatively small, it was comparable to other studies examining the effects of chemotherapy on gut microbiota [8,20,23], and considering feasibility, this sample size was calculated to produce adequate statistical power. However, as this was a small exploratory study, no controls for multiple comparisons for the correlation analyses were used, which increased the probability of type I error. As such, our results must be interpreted with caution and validated in larger cohorts.

Participants in our survivor group experienced diverse treatment regimens with respect to chemotherapy type, dose, and duration, in addition to other treatments such as surgery. This heterogeneity presents challenges with determining which potential aspects of cancer treatments may contribute to changes in the gut microbiota. Additionally, although colorectal cancers do frequently occur in young adults, patients with a colorectal cancer diagnosis were excluded from our sample, as research suggests changes in the gut microbiota are involved in the onset and/or progression of colorectal cancer [69,70]. As such, this would have presented a potential confounding variable within the analysis. We also decided to focus on a young adult cancer population in consideration of age-related differences that can occur in the gut microbiota [5]; thus, our results may not be generalizable to older adult cancer cohorts.

Additionally, while we originally planned to collect blood samples for cytokine and neurotransmitter analysis and to assess patients at three time points within the first 6 months post-treatment [40], this was no longer feasible due to pandemic-related challenges. This limited our capacity to evaluate other biomarkers and their potential associations with the gut microbiota and health outcomes. However, despite these limitations, we collected a rich data set with several outcomes that can be used to shape future, larger studies and to develop integrative interventions.

### 4.6. Implications and Future Directions

Knowledge of what bacterial species may be depleted after cancer treatments, such as chemotherapy, how long these perturbations may last, and the severity and degree of gut dysbiosis, GI and/or psychosocial symptoms is crucial to improving health outcomes in cancer survivors. Furthermore, clarifying potential mechanisms that affect GI symptoms and psychosocial health outcomes in cancer survivors will allow for tailored interventions to be developed. Although based on a small sample, the present findings can support the development of future, larger studies of a similar nature. Future studies may also be improved by investigating microbial metabolites and their potential role in GI and psychosocial symptoms. These findings may inform interventions for cancer survivors that aim to potentially prevent or reverse adverse GI and psychosocial symptoms following treatment by co-administering probiotics and employing other nutrition-based strategies.

Studies have shown that probiotic treatment may be particularly useful for GI symptoms during treatment, but confer greater benefits for psychosocial health after treatment has ended [71]. Moreover, based on our findings, the first 6 months to potentially 1 year post-treatment may be an especially critical time for microbiota-based interventions to be implemented. Developing integrative therapies to support both GI and psychosocial health via modulation of the gut microbiota could ultimately lead to a reduced symptom burden and improved quality of life in survivors of cancer.

## 5. Conclusions

Taken together, our findings suggest that survivors of cancer experience more GI symptoms and poorer psychosocial health compared to healthy peers. Time off treatment at less than 6 months was shown to result in lower alpha diversity, a potential indicator of gut microbiota dysbiosis. Moreover, significant differences were found in the relative abundance of specific microbes between survivors and controls, which correlated with specific psychosocial outcomes such as depressive functional interference and cognitive function. However, given that we did not control for multiple comparisons in this exploratory study, these results must be interpreted with caution. Nevertheless, the psychosocial symptoms reported by cancer survivors in our study may be, at least in part, influenced by changes in the gut microbiota that persist after treatments have ended. The first 6 months to potentially 1 year post-treatment may be an especially critical time to intervene using microbiota-based interventions.

## Figures and Tables

**Table 1:** Demographic characteristics of study participants (*N* = 35).

Demographic Variable		
	Survivors (*n* = 17)	Controls (*n* = 18)
Current Age in Years	M = 32.0 (±5.8)	M = 28.3 (±5.3)
	**% (*n*)**	**% (*n*)**
Sex		
Male	35.3 (6)	44.4 (10)
Female	64.7 (11)	55.6 (8)
Ethnicity		
Caucasian/white	58.8 (10)	77.8 (14)
First Nations	0	0
African American/Black	0	0
South Asian/Chinese/Filipino	5.9 (1)	11.1 (2)
Indian-subcontinent	11.8 (2)	5.6 (1)
Other	23.5 (4)	5.6 (1)
Education		
High school	11.8 (2)	5.6 (1)
Trade school	17.6 (3)	5.6 (1)
Community College	23.5 (4)	5.6 (1)
University (undergraduate)	35.3 (6)	50.0 (9)
University (professional/post-grad)	11.8 (2)	33.3 (6)
Income		
less than $30,000	29.4 (5)	38.9 (7)
$30,000 to less than $50,000	11.8 (2)	5.6 (1)
$50,000 to less than $80,000	41.2 (7)	11.1 (2)
$80,000 to less than $120,000	11.8 (2)	27.8 (5)
greater than $120,000	5.9 (1)	16.7 (3)
Marital Status		
Single (never legally married)	41.2 (7)	50.0 (9)
Married	35.3 (6)	16.7 (3)
Common-law	23.5 (4)	27.8 (5)
Divorced	0	0
Separated	0	5.6 (1)
Living Situation		
Living with a partner	58.8 (10)	55.6 (10)
Living with roommate(s)	11.8 (2)	5.6 (1)
Living with family	23.5 (4)	22.2 (4)
Living alone	5.9 (1)	16.7 (3)
Community		
Metropolitan	100 (17)	94.4 (17)
Regional/remote	0	5.6 (1)

**Table 2:** Clinical and treatment-related characteristics of survivors and controls.

Cancer Variables (Survivors *n* = 17)		
Age at Diagnosis (Years)	M = 30.1 (±6.0)	
	**% (*n*)**	
Cancer Type		
Breast	11.8 (2)	
Hodgkin Lymphoma	41.2 (7)	
Non-Hodgkin Lymphoma	23.5 (4)	
Sarcoma (bone and soft tissue)	11.8 (2)	
Other	11.8 (2)	
Cancer Stage		
I	23.5 (4)	
II	11.8 (2)	
III	17.6 (3)	
IV	29.4 (5)	
Unsure	17.6 (3)	
**Treatment Related Variables** (survivors *n* = 17)		
*Time off treatment (months)*	M = 16.9 (±16.4)	
*Time off treatment category*		
≤6 months	41.2 (7)	
>6 months	58.8 (10)	
Chemotherapy (yes)	100 (17)	
Surgery (yes)	35.3 (6)	
Radiation (yes)	17.6 (3)	
Hormone Therapy (yes)	11.8 (2)	
Immunotherapy (yes)	5.9 (1)	
Mucositis (during and/or after treatment) (yes)	23.5 (4)	
**Health Related Variables**	**Survivors (*n* = 17)** % (*n*)	**Controls** **(*n* = 18)** % (*n*)
Probiotics (within ≤2 years) (yes)	23.5 (4)	38.9 (7)
Antibiotics (within ≤2 years) (yes)	52.9 (9)	50.0 (9)
Corticosteroid medication (within ≤2 years) (yes)	23.5 (4)	5.6 (1)
*Smoking*		
No	94.1 (16)	72.2 (13)
Yes	0	11.1 (2)
Sometimes	5.9 (1)	16.7 (3)
Breastfed as infant (yes)	58.8 (10)	100 (18)
Birth delivery mode		
Vaginally	64.7 (11)	88.9 (16)
Caesarean section	29.4 (5)	11.2 (2)
Unsure	5.9 (1)	0
Body Mass Index (BMI)	M = 25.6 (±6.0)	M = 24.4 (±2.8)
Waist to Hip Ratio (WHR)	M = 0.85 (±0.1)	M = 0.82 (±0.1)
WHR risk category		
Low	41.2 (7)	88.9 (16)
Moderate	11.8 (2)	5.6 (1)
High	17.6 (3)	0
Missing	29.4 (5)	5.6 (1)

**Table 3:** Associations between alpha diversity and psychosocial outcomes.

Psychosocial Outcomes	Chao1	Shannon Index
Anxiety	*r* = −0.11, *p* = 0.53	*r* = −0.12, *p* = 0.51
Depressive symptoms	*r*= −0.13, *p*= 0.45	*r* = −0.15, *p* = 0.40
Depressive functional	***r* = −0.47, *p* = 0.004**	***r* = −0.39, *p* = 0.022**
PTSD symptoms	*r* = −0.18, *p* = 0.39	*r* = −0.18, *p* = 0.41
Fatigue	*r* = 0.10, *p* = 0.57	*r* = 0.13, *p* = 0.44
Social isolation	*r* = −0.09, *p* = 0.61	*r* = −0.08, *p* = 0.67
Pain behavior	*r* = −0.23, *p* = 0.60	*r* = −0.17, *p* = 0.50
Cognitive function	***r* = 0.34, *p* = 0.048**	***r* = 0.33, *p* = 0.05**

Note. Significant associations (*p* < 0.05) in bold.

## Data Availability

Data are available upon reasonable written request to the corresponding author.

## Supplementary Materials

The following supporting information can be downloaded at: https://www.mdpi.com/article/10.3390/curroncol29050243/s1, Figure S1: Alpha diversity on the Shannon Index in cancer survivors and healthy controls; Figure S2: Heatmap of correlations between psychosocial outcomes and ASVs at the order (o), family (f), and genus (g) levels in healthy controls; Figure S3: Heatmap of correlations between GI outcomes and ASVs at the order (o), family (f), and genus (g) levels in healthy controls.

## Abbreviations

Gastrointestinal (GI); Lipopolysaccharide (LPS); Post-traumatic stress disorder (PTSD); Attention deficit hyperactivity disorder (ADHD); Body Mass Index (BMI); Waist-to-Hip Ratio (WHR); Remote Electronic Data Capture (REDCap); Electronic Data Capture (EDC); Clinical Research Unit (CRU); National Institutes for Health (NIH); Patient-Reported Outcomes Measurement Information System (PROMIS); Amplicon sequence variants (ASV); Permutational multivariate analysis of variance (PERMANOVA); Analysis of Variance (ANOVA); Standard Error of the Mean (SEM); Confidence Interval (C.I.); Statistical Package for the Social Sciences (SPSS); Mean (M); Standard Deviation (SD); Irritable Bowel Syndrome (IBS); Fear of cancer recurrence (FCR); Hypothalamic-Pituitary-Adrenal (HPA).
