# Supplementary material for: The Chemo-Gut Pilot Study: Associations between Gut Microbiota, Gastrointestinal Symptoms, and Psychosocial Health Outcomes in a Cross-Sectional Sample of Young Adult Cancer Survivors
Source: Curr Oncol. 2022 Apr 21;29(5):2973–94. doi: 10.3390/curroncol29050243 (PMC9140183; doi:10.3390/curroncol29050243)
Supplement: Supplementary file 1 [file curroncol-29-00243-s001.zip › curroncol-1665490-supplementary.pdf]

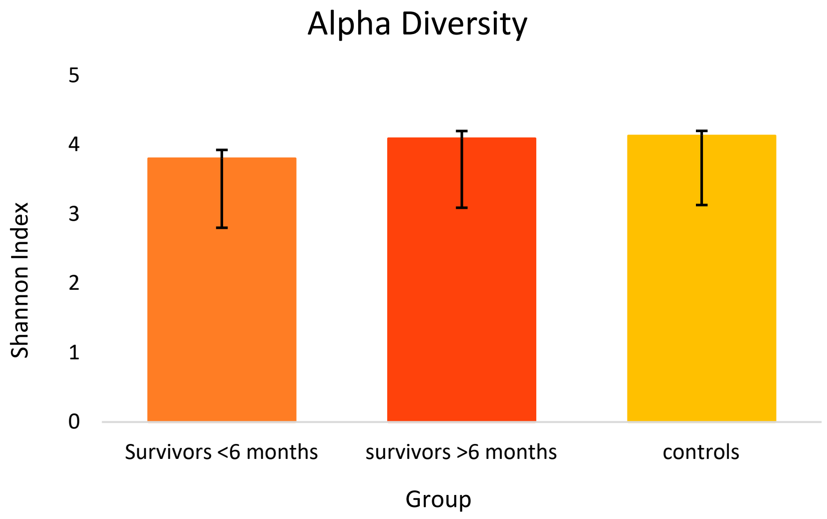

Supplementary Figure (S1). Alpha diversity on the Shannon Index in cancer survivors and healthy controls. Group means are presented. Error bars represent standard error of the mean (SEM). Group means were not statistically significantly different. Group n's = 7 (<6 months), 10 (>6 months), and 18 (controls).

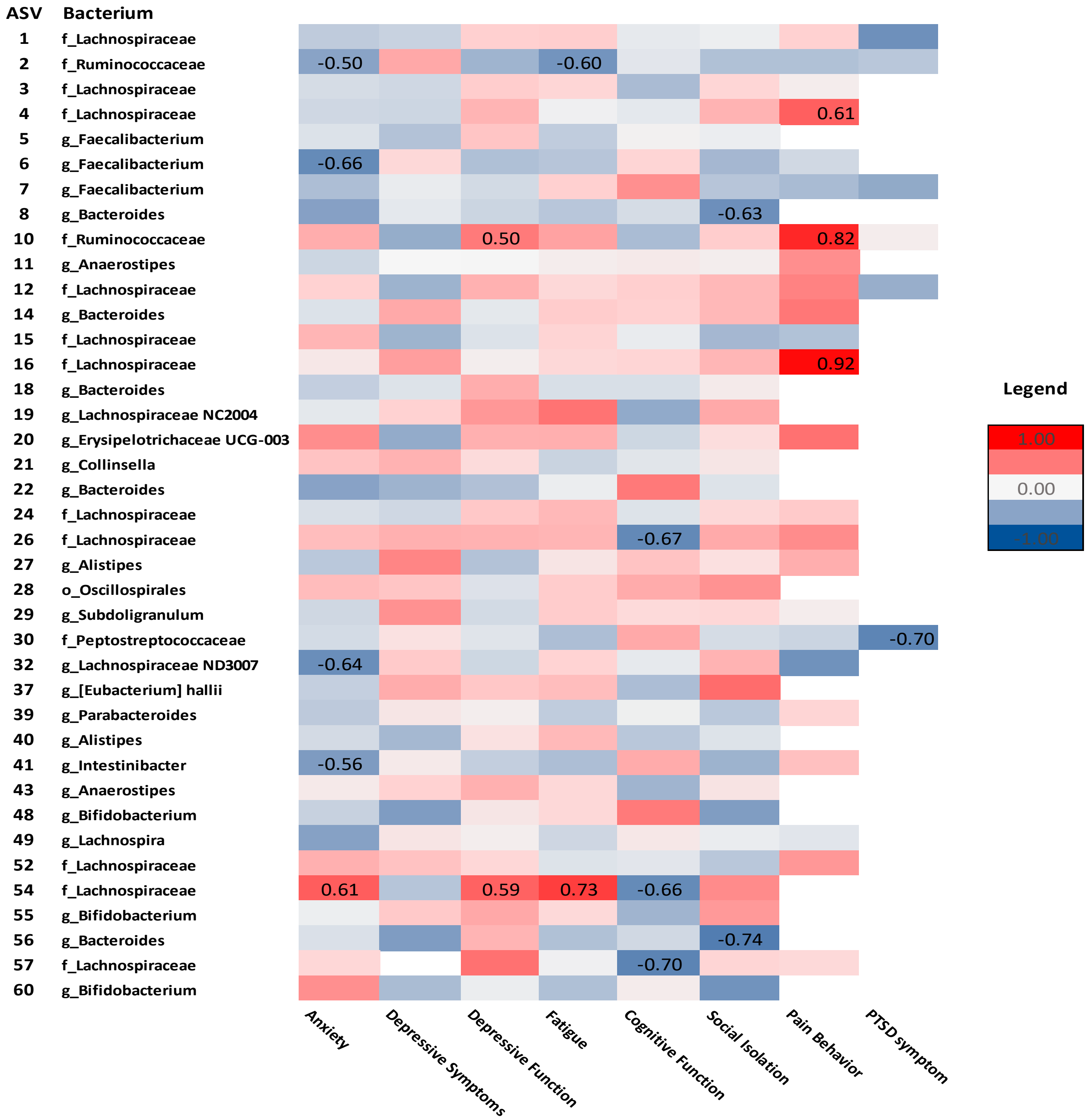

Supplementary Figure (S2). Heatmap of correlations between psychosocial outcomes and ASV's at the order (o), family (f), and genus (g) levels in healthy controls. Spearman's rho is presented. *Ruminococcaceae* (ASV_2) (rho= -.50, *p*= .04), *Faecalibacterium* (ASV_6) (rho= -.66, *p* =.01), *Lachnospiraceae ND3007* (ASV_32) (rho= -.64, *p*= .011), *Intestinibacter* (ASV_41) (rho= -.56, *p*= .05), and *Lachnospiraceae* (ASV_54) (rho= .61, *p*= .03) all correlated with anxiety. *Ruminococcaceae* (ASV_2) (rho= -.60, *p*= .01) and *Lachnospiraceae* (ASV_54) (rho= .73, *p*= .01) both correlated with fatigue. *Ruminococcaceae* (ASV_10) (rho= .50, *p*= .05) and *Lachnospiraceae* (ASV_54) (rho= .59, *p*= .04) both correlated positively with depressive functional interference. *Lachnospiraceae* (ASV's 26, 54, and 57) all correlated negatively with

cognitive function (rho= -.66 – -.70, *p*< .05). *Bacteroides* (ASV's 8 and 56) both correlated negatively with social isolation (rho= -.63 – -.74, *p*< .05). *Lachnospiraceae* (ASV's 4 and 16) (rho= .61 - .92, *p*< .05) and *Ruminococcaceae* (ASV_10) (rho= .82, *p*= .004) all correlated positively with pain behaviour. *Peptostreptococcaceae* (ASV_30) correlated negatively with PTSD symptoms (rho= -.70, *p*= .03). (n's range from 10 – 18).

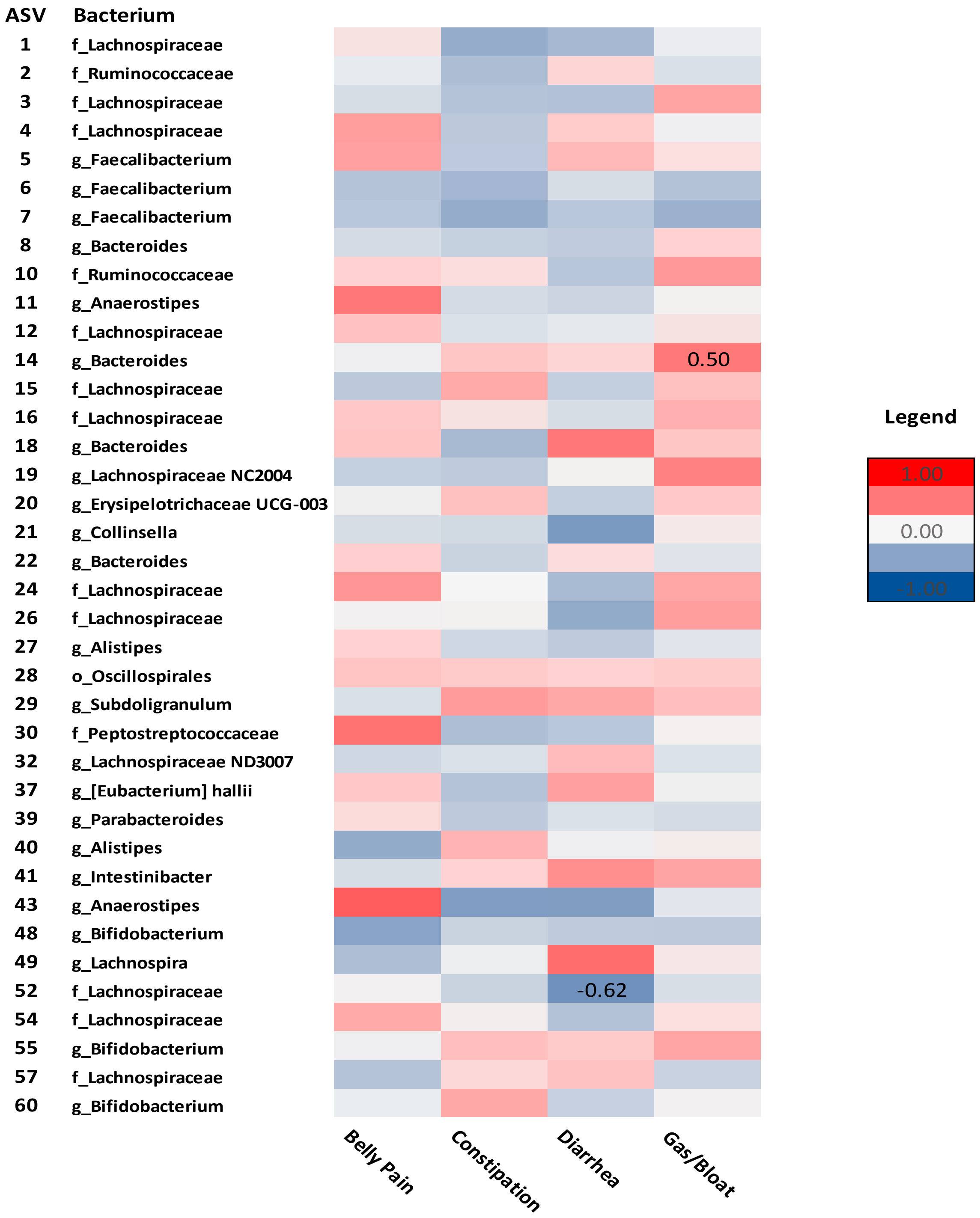

Supplementary Figure (S3). Heatmap of correlations between GI outcomes and ASV's at the order (o), family (f), and genus (g) levels in healthy controls. Spearman's rho is presented. *Bacteroides* (ASV_14) correlated with gas and bloating (rho= .50, *p*= .05), and *Lachnospiraceae* (ASV_52) with diarrhea (rho= -.62, *p*= .01). (n's range from 10 – 18).
